# NLRP3 inflammasome: A link between systemic infection and Alzheimer’s disease

**DOI:** 10.4103/NRR.NRR-D-25-00073

**Published:** 2025-06-19

**Authors:** Tatiana Barichello, Felipe Dal-Pizzol

**Affiliations:** Department of Psychiatry and Behavioral Sciences, McGovern Medical School, The University of Texas Health Science Center at Houston (UTHealth), Houston, TX, USA; Laboratory of Experimental Pathophysiology, Graduate Program in Health Sciences, University of Southern Santa Catarina (UNESC), Criciúma, SC, Brazil

Neuroinflammation is a crucial factor in the progression of various diseases, ranging from immune-related conditions such as sepsis to neurodegenerative disorders such as Alzheimer’s disease (AD) (Ravichandran and Heneka, 2024). This perspective article, which draws on insights from diverse fields including neuroscience, immunology, and pathology, provides a critical analysis of ongoing research efforts in inflammasome biology, with specific emphasis on Nod-like receptor (NLR) and pyrin domain-containing protein 3 (NLRP3). This article takes an interdisciplinary approach, placing emphasis on the relationship between sepsis and AD, in an attempt to advance our understanding of these multifaceted diseases and the role of infection on neuroinflammation and AD pathology.

**Figure 1:**
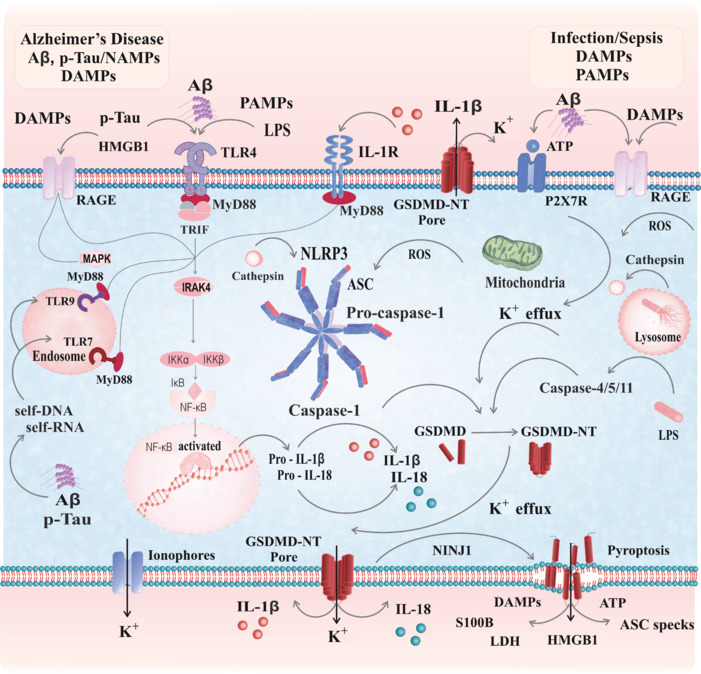
NLRP-3 inflammasome activation in neuroinflammation and Alzheimer’s disease. This figure illustrates the complex pathways involved in microglial reactivity during neuroinflammation and pyroptosis, highlighting the role of inflammasome activation triggered by various DAMPs, NAMPs, and PAMPs. DAMPs such as Aβ, p-Tau, and HMGB1, along with PAMPs such as lipopolysaccharide, interact with receptors such as TLR4 and IL-1R on the cell membrane, initiating signaling cascades. These cascades activate NF-κB via TLRs, including TLR4, TLR7, and TLR9, leading to the transcription of proinflammatory cytokines, such as pro-IL-1β and pro-IL-18. The NLRP3 inflammasome is activated through signals such as potassium (K^+^) efflux, reactive oxygen species, and lysosomal disruption, forming a complex with ASC and pro-caspase-1. Caspase-1 then processes pro-IL-1β and pro-IL-18 into active cytokines, which are released to propagate inflammation. Additionally, caspase-1, along with caspase-4/5/11, cleaves gasdermin D (GSDMD), generating GSDMD-NT fragments that create pores in the cell membrane. Subsequently, NINJ1-mediated membrane rupture contributes to a sustained inflammatory environment that can exacerbate neuronal damage and progression of the disease, facilitating cytokine release and K^+^ efflux. This process leads to pyroptosis, a form of inflammatory cell death, and the release of secondary DAMPs such as HMGB1, ATP, LDH, and S100B, further amplifying inflammation (CorelDRAW™). Aβ: Amyloid-beta; ASC: apoptosis-associated speck-like protein containing a CARD; ATP: adenosine triphosphate; DAMPs: danger-associated molecular patterns; GSDMD: gasdermin D; GSDMD-NT: gasdermin D N-terminal fragment; HMGB1: high mobility group box 1; IL: interleukin; LDH: lactate dehydrogenase; LPS: lipopolysaccharide; NAMPs: neurodegeneration-associated molecular patterns; NF-κB: nuclear factor kappa B; NINJ1: ninjurin-1; NLRP3: Nod-like receptor (NLR) and pyrin domain-containing protein 3; PAMPs: pathogen-associated molecular patterns; p-Tau: phosphorylated Tau; ROS: reactive oxygen species; TLR: Toll-like receptor.

The NLRP3 inflammasome is a multi-subunit protein complex that acts as a cytosolic pattern recognition receptor. The canonical NLRP3 inflammasome pathway follows a two-step activation process: priming and activation. During priming, cytokine receptors (e.g., interleukin-1 receptor [IL-1R] and toll-like receptor 4 [TLR4]) interact with ligands, recruiting myeloid differentiation primary response 88 adaptor proteins. This secondary messenger activation leads to kinase activation, including inhibition of nuclear factor kappa-B kinase (IκB kinase), which phosphorylates and degrades the inhibitor of nuclear factor kappa-B (NF-κB), a suppressor of NF-κB. This degradation releases NF-κB, enabling its movement into the nucleus, where it initiates the transcription of pro-interleukin-1 beta (pro-IL-1β), pro-IL-18, and NLRP3 inflammasome components. Then, in the activation step, the NACHT protein domain is oligomerized which promotes the assembly of the inflammasome. The NACHT protein is named after the proteins where it was first identified, including NLR family apoptosis inhibitory protein, Class II major histocompatibility complex transactivator, heterokaryon incompatibility protein (HET-E), and telomerase-associated protein 1. The NACHT domain oligomerization allows NLRP3 molecules to self-associate and bring in a pyrin domain located at the N-terminus. This allows the NLRP3 to connect with apoptosis-associated speck-like proteins containing a caspase recruitment domain. Subsequently, the CARD domain of ASC binds to the CARD domain of pro-caspase-1, triggering an auto-activation through proximity-induced dimerization and cleavage into active caspase-1, which processes pro-IL-1β and pro-IL-18 into their biologically active forms. Caspase-1 activation also cleaves gasdermin D (GSDMD), generating its N-terminal fragment (GSDMD-NT). The GSDMD-NT forms pores in the plasma membrane, facilitating the release of IL-1β, IL-18, and ion flux. Furthermore, the formation of GSDMD-NT pores triggers the rupture of the plasma membrane and it is mediated by nerve injury-induced protein 1 (ninjurin-1, NINJ1). This leads to the release of various damage-associated molecular patterns (DAMPs) into the extracellular environment, including high-mobility group box-1 (HMGB1), lactate dehydrogenase, and S100B. In the non-canonical pathway, guanylate-binding proteins detect membrane-bound vacuoles of invading gram-negative bacteria, enabling the release of cytosolic lipopolysaccharide that interacts with caspases 4, 5, or 11, which then undergo auto-activation. The active forms of caspases-4/5/11 cleave GSDMD, leading to pyroptosis and triggering the NLRP3 inflammasome by promoting intracellular potassium (K⁺) efflux. In the alternative NLRP3 pathway, stimulation of TLR4 activates NLRP3 via the TIR-domain-containing adapter-inducing interferon-β adaptor protein. The precise mechanism, although unclear, involves receptor-interacting protein kinase 1, Fas-associated protein with death domain, and caspase-8 (Vande Walle and Lamkanfi, 2024). This process is a crucial driver of the chronic inflammation seen in AD and the acute inflammatory response in sepsis; refer to **Figure 1** for more details.

AD is a neurodegenerative disease that progressively worsens over time and is the principal cause of dementia among older adults. Its hallmark pathological features include the accumulation of amyloid-beta (Aβ) plaques and hyperphosphorylated tau (p-Tau) tangles. These aggregates act as DAMPs, triggering an immune response by activating pattern recognition receptors, such as TLRs. This activation primes microglia, resident immune cells in the brain, and initiates processes such as phagocytosis, contributing to the neuroinflammatory cascade associated with AD progression (Ravichandran and Heneka, 2024; Vande Walle and Lamkanfi, 2024). Microglia recognize Aβ aggregates through specific cell surface receptors, such as TREM2, CD36, and scavenger receptors. These receptors bind to Aβ, initiating the phagocytic process. Once Aβ is internalized in the microglia phagosome, it fuses with early endosomes. Then late endosome forms a phagolysosome, which contains hydrolytic enzymes including proteases, lipases, and nucleases. In AD, the disruptions of lysosomal function, impaired phagosome maturation, or receptor signaling issues can impair the ability of microglia to degrade Aβ efficiently, causing microgliopathy. Furthermore, the incomplete phagocytosis of Aβ and p-Tau interferes with the electron transport chain, specifically affecting complexes I and III, leading to free electron leakage which reacts with oxygen to form superoxide and other reactive oxygen species.

As a consequence, damaged mitochondria can release mitochondrial DNA, cardiolipin (phospholipid of the inner mitochondrial membrane), proteins, and lipids components that, when released into the cytoplasm, lead to Aβ accumulation within the microglia or its release back into the extracellular space, contributing to NLRP3 activation, a chronic neuroinflammation process leading to a brain plaque formation (Ravichandran and Heneka, 2024). In mouse models of AD, including those characterized by amyloidosis (Venegas et al., 2017) or tauopathy (Ising et al., 2019), NLRP3 inflammasome expression has been observed in the brain (Venegas et al., 2017; Ising et al., 2019). Comparable observations have been documented in postmortem brain tissue from individuals with AD, reinforcing its role in the underlying disease pathology (Vontell et al., 2023).

Following NLRP3 activation, cells may undergo pyroptosis, a form of inflammatory cell death. During pyroptosis, the cell membrane ruptures, releasing ASC specks and other cellular contents into the extracellular environment (Hu et al., 2024). Once outside the cell, extracellular ASC specks drive inflammation by acting as DAMPs, activating surrounding microglia and other immune cells and amplifying the inflammatory response. Additionally, ASC specks can act as “seeds” for further aggregation, binding to Aβ and p-Tau, promoting the formation of amyloid plaques and tauopathy with a hallmark of AD (Lobanova et al., 2024). Recently, ASC specks have been measured in blood and cerebrospinal fluid as biomarkers of inflammation and inflammasome activity in Parkinson’s disease and AD. High diagnostic accuracies were achieved by calculating ratios that combine ASC speck levels with Aβ, α-synuclein, and p-Tau aggregates in serum. Specifically, a (p-Tau-AT8 + ASC)/Aβ ratio yielded diagnostic accuracies of 92% for early AD and 95% for late AD, while both (α-syn + ASC)/Aβ and ASC/Aβ ratios reached up to 98% accuracy for Parkinson’s disease. These support the hypothesis that the NLRP3 inflammasome is activated in the early stages of neurodegenerative diseases (Lobanova et al., 2024).

The growing evidence from *in vitro* studies, animal models, and postmortem brain tissue analyses indicates that systemic inflammation and or infection can trigger neuroinflammation and activate inflammasomes, ultimately contributing to sepsis-associated encephalopathy. Sepsis is a life-threatening medical condition due to a dysregulated host immune response to infection, resulting in widespread inflammation, tissue damage, and organ dysfunction. In preclinical studies, serum markers in Wistar rats subjected to cecal ligation and perforation, a gold standard model for studying sepsis, demonstrated features indicative of the acute proinflammatory phase of sepsis. This included a rapid increase in tumor necrosis factor-α, IL-1β, and IL-6, accompanied by a progressive rise in ligands for the receptor for advanced glycation end-products (RAGE), such as S100B, Nϵ-[carboxymethyl]lysine, HSP70, and HMGB1. Concurrently, in the brain, there was an elevation in RAGE and TLR-4, glial fibrillary acidic protein, neuronal nitric oxide synthase, and the pathological markers Aβ and p-TauSer-202 (Gasparotto et al., 2018). Additionally, APP/PS1 transgenic mice subjected to sepsis revealed an accelerated and aggravated progression of AD pathology, accompanied by notable reductions in short-chain fatty acids, alterations in gut microbiota α- and β-diversity, reduced intestinal villus height and crypt depth, and an increased presence of microglial cells in proximity to Aβ plaques (Giridharan et al., 2023). Blocking RAGE (Gasparotto et al., 2018), NLRP3 (Danielski et al., 2020; Dominguini et al., 2022), or its underlying pathway rescued memory in sepsis (Danielski et al., 2020; Giridharan et al., 2023) and in AD (Lonnemann et al., 2020) mice models. These results suggest a strong interplay between systemic infection (Gasparotto et al., 2018; Giridharan et al., 2023), gut dysbiosis (Giridharan et al., 2023), and neuroinflammation in underlying mechanisms of AD pathology (Gasparotto et al., 2018; Danielski et al., 2020; Lonnemann et al., 2020; Giridharan et al., 2023).

In conclusion, activation of the NLRP3 inflammasome is crucial in driving neuroinflammation and serves as a common mechanistic pathway linking systemic inflammation, infection, and neurodegenerative diseases, including AD. This inflammasome pathway facilitates the inflammatory response and may act as a bridge through which infections accelerate AD pathology. Through the release of proinflammatory cytokines, NLRP3 activation amplifies neuroinflammatory signals that exacerbate neuronal damage, thereby contributing to the progression of AD and potentially other neurodegenerative disorders.

*This work was supported by Texas Alzheimer’s Research and Care Consortium – TARCC 2022-26, The National Football League Players Association - NFLPA, NIH/NIA Grant 1R01 AG072491 to TB and FDP.*
